# Characteristics of Blood Metabolic Profile in Coronary Heart Disease, Dilated Cardiomyopathy and Valvular Heart Disease Induced Heart Failure

**DOI:** 10.3389/fcvm.2020.622236

**Published:** 2021-01-20

**Authors:** Chang Liu, Ruihua Li, Yang Liu, Zhenguo Li, Yujiao Sun, Peiyuan Yin, Rihong Huang

**Affiliations:** ^1^First Affiliated Hospital of Dalian Medical University, Dalian, China; ^2^Medical Laboratory Science, Second Affiliated Hospital of Dalian Medical University, Dalian, China; ^3^College of Integrative Medicine, Dalian Medical University, Dalian, China

**Keywords:** heart failure, LC-MS, metabolomics, biomarker, metabolism

## Abstract

**Purpose:** Metabolic impairment is one key contributor to heart failure (HF) pathogenesis and progression. The major causes of HF, coronary heart disease (CHD), dilated cardiomyopathy (DCM), and valvular heart disease (VHD) remains poorly characterized in patients with HF from the view of metabolic profile. We sought to determine metabolic differences in CHD-, VHD-, and DCM-induced HF patients and identify significantly altered metabolites and their correlations.

**Procedure:** In this study, a total of 96 HF cases and 97 controls were enrolled. The contents of 23 amino acids and 26 carnitines in fasting plasma were measured by a targeted liquid chromatography and mass spectrometry (LC-MS) approach.

**Results:** Nine metabolites (Histidine, Arginine, Citrulline, Glutamine, Valine, hydroxyhexadecenyl-carnitine, acylcarnitine C22, hydroxytetradecanoyl-carnitine, and carnitine) were found to be related with the occurrence of HF. Arginine, Glutamine and hydroxytetradecanoyl-carnitine could effectively distinguish CHD and DCM patients, and hydroxytetradecanoyl-carnitine and aspartic acid were able to classify CHD and VHD cohorts.

**Conclusion:** This study indicated that circulating amino acids and long-chain acylcarnitine levels were closely associated with progression of heart failure. Monitoring these metabolic alterations by LC-MS may help the differentiation of CHD, VHD, and DCM in the early stage, and provide new diagnostics targets or therapeutic interventions.

## Introduction

Cardiovascular disease remains to be an enormous clinical and economic burden worldwide. Due to the progressive aging of the general population and improved treatment strategies for ischemic heart disease and myocardial infarction (1), heart failure (HF) prevalence is increasing; furthermore, the morbidity and mortality of HF are still unacceptably high. A prominent obstacle to therapeutic development is the poor understanding of HF pathogenesis and pathophysiology.

Coronary heart disease (CHD), dilated cardiomyopathy (DCM) and valvular heart disease (VHD) are the three most common causes of HF. The clinical picture of CHD is defined by an atherosclerotic plaque-induced narrowing of the coronary arteries, which results in myocardial ischemia, infarction, and post-infarction HF. DCM may arise as a result of structural and functional myocardial abnormalities that are characterized by systolic dysfunction and dilation of the left and/or right ventricles (2, 3). VHD is a multifactorial and complex disorder due to pathological remodeling of the valve tissues, as well as secondary consequences from a change in the anatomy and geometry of cardiac structures (4). Although CHD, DCM, and VHD have different origins, they each lead to HF. Metabolomics has been widely used in HF mechanistic investigation, and cardiac metabolic impairment is confirmed to be mainly associated with global suppression of metabolic fuel intake (glucose, fatty acid, and amino acids) (5–9). In brief, energy production in cardiomyocytes is shifted from fatty acids toward glycosis, anaplerosis, and other forms of metabolism, such as use of lactate, branched-chain amino acids, and ketone bodies (10–12), which may not able to compensate for the reduced fatty acid and glucose oxidation and result in energy deficiency and the development of HF.

A considerable body of evidence suggests that myocardial substrate and energy metabolism play a key role in affecting the development and prognosis of HF (10, 11, 13). Additionally, the metabolic state of the heart is closely related to the intricate signaling network and transcription factors controlling gene expression (14, 15). Various studies have explored the plasma metabolites of HF patients as potential biomarkers (7, 16–18), but few focused on the metabolic profile differences among CHD, DCM, and VHD, which may be vital for effective disease management. Here, we report a metabolomics study of clinical plasma samples to characterize the specific metabolic profiles of CHD, DCM, and VHD patients. Our study aims to advance our understanding of the pathophysiology of HF from metabolic view and better distinguish it among CHD-, VHD-, and CDM-induced HF, thus potentially supplementing the early diagnosis of the disease.

## Materials and Methods

### Study Population and Sample Collection

Clinical data were extracted from the medical records of patients from the First Affiliated Hospital of Dalian Medical University, Liaoning, China. Between December 2018 and December 2019, 119 consecutive patients and 97 normal individuals were enrolled in this study. Individuals enrolled were aged 18–90 years. Inclusion criteria conforms to the diagnostic criteria of Framinghan heart failure, New York Heart Association (19) level III-IV. Heart failure is defined as follows: clinical manifestations, including cardiac enlargement and decreased ventricular systolic function with or without congestive heart failure, which are often accompanied by arrhythmia, embolism, sudden death, and other complications; enlarged X-ray of the heart showing a Bosom ratio > 0.5, and ultrasonic echocardiography showing that the whole heart was enlarged, especially the left ventricle; and a decrease in ventricular systolic function, a decrease in the diffuse motion of the wall of the chamber detected by echocardiography, and the ejection fraction being lower than normal. Exclusion criteria are as follows: perinatal cardiomyopathy; alcoholic cardiomyopathy; cardiomyopathy caused by metabolic and internal secretory disease, hyperthyroidism, amyloidosis, or diabetes; cardiomyopathy caused by hereditary familial neuromuscular disorders; cardiomyopathy caused by systemic diseases such as systemic lupus erythematosus, rheumatoid arthritis, etc.; and toxic cardiomyopathy. In this study, VHD was composed of rheumatic valvular disease and primary senile valvular disease, which were all confirmed by surgery and pathological results, suggesting mucoid degeneration and calcification.

This study was approved by the Ethics Review Committee of the First Affiliated Hospital of Dalian Medical University. The blood samples were obtained from all participants after the initial presentation. Sample transfer, centrifugation, and separation were completed within 1 h to avoid any pre-analytical factors that may affect plasma metabolite stability. Samples were stored at −80°C until they were analyzed.

### Targeted LC-MS/MS Analysis

#### Chemicals and Reagents

HPLC grade acetonitrile was purchased from Merk (Merk, Darmstadt, Germany). Pure water was purified by a Milli-Q system (Millipore, Billerica, MA, USA). N-butanol, acetyl chloride, amino acids and carnitines were purchased from Sigma-Aldrich (St. Louis, MO, USA). Isotopic internal standards were purchased from Cambridge Isotope Laboratories (Tewksbury, MA, USA), and were all mixed and dissolved in 2 mL pure methanol and stored at 4°C. The working fluid was diluted 100 times to extract metabolites.

#### Sample Processing

A circle 3 mm diameter from each dried blood spot was made. Then, 3 mm-blood-stains were placed in Millipore multilayer 96-well plates (Millipore, Billerica, MA, USA) for metabolite extraction. Every 100 μL of working fluid was added to the hole containing the dried blood spot paper to make a circle with a diameter of 3 mm. After 20 min of slight oscillation, the 96-well plate was centrifuged at 1,500 g for 2 min, and filtrate collected through the lower layer of the 96-well plate. Four blank holes were randomly selected from each plate, and two low controls and two high controls were placed separately. QC and filtrate were blow-dried with pure nitrogen at 50°C. The dried samples were derived with a mixture of 60 μl acetyl chloride and n-butanol (volume ratio 1:9) at 65°C for 20 min. Then, the derived samples were dried using the method mentioned previously. Each dry sample was dissolved with 100 μl fresh mobile phase solution for metabolite analysis.

#### Metabolites Analysis

The AB Sciex 4000 QTrap system (AB Sciex, MA, USA) was used for the direct sampling mass spectrometry metabolites analysis. The equipped ion source is an electrospray ion source that scans all analytes in positive ion mode. The injection volume was 20 μl, with mobile phase of 80% acetonitrile aqueous solution, and initial flow rate of 0.2 mL/min. Then, the flow rate decreased to 0.1 mL/min within 0.08 min, remained constant until 1.5 min, returned to 0.2 mL/min within 0.01 min, and remained unchanged for another 0.5 min. The ion spray voltage was 4.5 kv, the curtain gas pressure was set to 20 psi, the ion source gas 1 and gas 2 were set to 35 psi, and the auxiliary heating temperature was set to 350°C. The Analyst v1.6.0 software (AB Sciex) was used to control the system and collect data.

#### Data Processing and Statistical Analysis

MetaboAnalyst 4.0 (http://www.metaboanalyst.ca/; Wishart Research Group, McGill University, Canada) was used for outlier identification and to assess quality, homogeneity, and dominating trends of the group separation inherent in the data set with unsupervised principal component analysis (PCA). Additionally, it was used to distinguish between the classes, to identify the differential metabolites with partial least squares discriminant analysis (PLS-DA), and to generate variable importance in the projection (VIP) value of the PLS-DA model. Student's *t*-test or Mann–Whitney test were performed to determine the difference in metabolites between the two groups. One-way analysis of variance (20) or Kruskal–Wallis test were used with three or more groups of continuous variables. Metabolites with VIP > 1 and *p* < 0.05 were considered to be the most likely metabolites that could be used to analyze the differences between the two groups and to assess the severity of HF. Participant data were averaged and then normalized to generate a heat map using the heatmap package (R version 3.6) and correlation analysis in Galley package (R version 3.6). The receiver operating characteristic (ROC) and the respective area under the ROC curve (AUC) were calculated using the ROC package of the R software (version 3.6). ROC curve analysis estimated the optimal cut-off values maximizing sensitivity and specificity between low and high levels of methylation. The statistical analysis was performed on GraphPad Prism 8.0. The results with *p* < 0.05 were considered statistically significant.

## Results

### Baseline Characteristics of the Study Population

**Figure 1:**
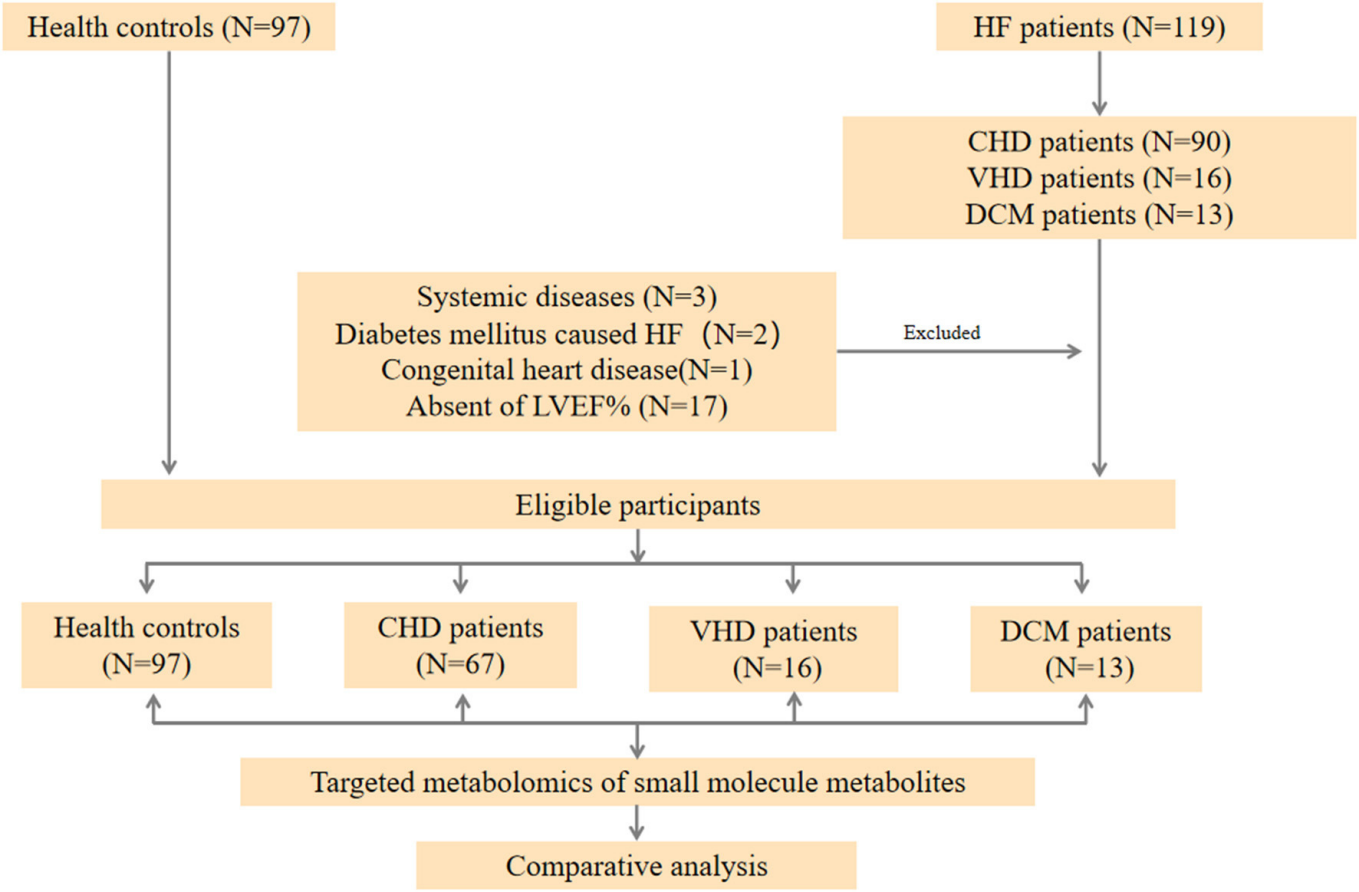
Workflow of the present study. In general, HF patients with different etiology (67 CHD patients, 16 VHD patients, and 13 DCM patient, respectively), and 96 healthy controls were enrolled in our study. A targeted metabolomics panel was applied to the plasma of these individuals. We comparatively analyzed the plasma metabolomics data of the four groups to elucidate the differences among these patients with different etiology.

Targeted metabolomics technique was used to assess the plasma samples. Briefly, 119 HF patients were consecutively enrolled in the First Affiliated Hospital of Dalian Medical University and patients with the diagnosis of CHD, VHD, or DCM were selected. Twenty three patients were excluded according to the exclusion criteria. 67 CHD patients, 16 VHD patients, and 13 DCM patients were finally selected for further study (Figure 1). Table 1 represents the baseline clinical characteristics of the study population. Median and interquartile (IQR) ranges for each cohort were measured. When compared to the control group, HF cases (CHD, VHD, and DCM) were more likely to have higher B-type natriuretic peptide (BNP, pg/mL: 1819.07/3320.00/4398.38 vs. 42.60), blood urea nitrogen (BUN, mmol/L:11.07/12.58/7.33 vs. 5.60), creatinine (CRE, mmol/L: 115.00/105.00/145.00 vs. 63.50), and lower cholesterol (Chol, mmol/L: 3.88/4.04/3.91 vs. 4.94), triglyceride (TG, mmol/L: 1.12/1.07/1.15 vs. 1.47), high-density lipoprotein (HDL, mmol/L: 0.99/0.87/0.93 vs. 1.34), low-density lipoprotein (LDL, mmol/L: 2.06/2.43/2.74 vs. 2.70). These characteristics were in alignment with those from major epidemiological studies, thus supporting the generalizability of our HF and control cohorts to broader populations (21–23).

**Table 1:** Baseline Characteristic of the participants.

**Variable**	**HF**			**Control** ** (*n* = 97)**	***P*-value**
	**CHD** ** (*n* = 67)**	**VHD** ** (*n* = 16)**	**DCM** ** (*n* = 13)**		
**Baseline**					
Age (yrs)	78 (70.75–78)	66 (60–73.25)	49 (27–45)	62 (55–65)	0.477
Gender (male)	33 (49.25%)	8 (50%)	7 (53%)	42 (43.30%)	0.782
SBP (mmHg)	120.5 (108.5–120)	122.5 (103.5–146.25)	112 (87.5–111)	–	0.253
DBP (mmHg)	70.5 (61.25–70)	69.5 (66.5–72.5)	65.5 (53.75–64)	–	0.685
HR (beats/min)	85 (76–85)	81 (76.5–93.75)	85.5 (73.25–84)	–	0.906
**Echocardiography**					
LAD (mm)	57 (50–57)	58 (45–62)	66 (60–77)	–	
LVEF%	35 (28–35)	40 (33–57)	24 (19–26)	–	
**Serum biomarkers**					
BNP (pg/mL)	1819.07 (819.76–1819.07)	3320 (1270.15–4275.32)	4398.38 (1789–8976.96)	42.60 (16.3–61.7)	0.085
BUN (mmol/L)	11.07 (7.51–11.07)	12.58 (6.31–17.38)	7.33 (5.06–13.23)	5.6 (5.08–6.64)	0.767
CRE (μmol/L)	115 (83–115)	105 (59–140)	145 (92–147)	63.53 (50.58–73.75)	0.661
Tn (μg/L)	1.84 (0.136–1.84)	0.09 (0.06–0.21)	0.12 (0.04–0.17)		0.001
Albumin (g/L)	35.75 (32.53–35.7)	36.5 (29.35–37.9)	34.4 (31.1–32.95)	–	0.591
CysC (mg/mL)	1.96 (1.28–1.99)	0.63	0.87 (0.79–1.03)	0.94 (0.79–1.08)	0.002
Chol (mmol/L)	3.88 (3.16–3.88)	4.04 (3.82–4.37)	3.91 (3.54–4.31)	4.94 (4.43–5.64)	0.910
TG (mmol/L)	1.12 (0.84–1.12)	1.07 (0.87–1.23)	1.15 (1.02–1.3)	1.47 (1.05–2.22)	0.850
HDL (mmol/L)	0.99 (0.79–0.99)	0.87 (0.74–1.00)	0.93 (0.78–0.98)	1.34 (1.1–1.5)	0.465
LDL (mmol/L)	2.06 (1.65–2.06)	2.43 (2.30–2.58)	2.74 (2.18–2.76)	2.70 (2.32–3.35)	0.704
**Comorbidity**					
Hypertension	46 (68.66%)	7 (43.75%)	3 (23.07%)		0.017
Diabetes	37 (55.22%)	6 (37.5%)	2 (15.38%)		0.054
Atrial fibrillation	19 (28.36%)	11 (68.75%)	2 (15.38%)		0.024
**Medication**					
ACEI/ARBs	28 (41.79%)	7 (43.75%)	5 (38.46%)		0.991
Beta-blockers	62 (92.54%)	13 (81.25%)	12 (92.31%)		0.308
Spironolactone	30 (44.78%)	7 (43.75%)	11 (84.61%)		0.263
Trimetazidine	25 (37.31)	2 (12.5%)	2 (15.38%)		0.184
Statin	53 (79.10%)	7 (43.75%)	2 (15.38%)		<0.001
Aspirin	61 (91.04)	6 (37.5%)	2 (15.38%)		<0.001

*Values are reported as median ± interquartile range or as number (%)*.

*SBP, systolic blood pressure; DBP, diastolic blood pressure; HR, heart rate; LAD, left atrium diameter; LVEF, left ventricular ejection fraction; BNP, B-type natriuretic peptide; BUN, blood urea nitrogen; CRE, creatinine; Tn, troponin; CysC, cystatin C; Chol, cholesterol; TG, Triglyceride; HDL, high-density lipoprotein; LDL, low-density lipoprotein; ACEI/ARBs, angiotensin-converting enzyme inhibitor/angiotensin receptor blockers*.

### Metabolites Associated With Heart Failure

**Figure 2:**
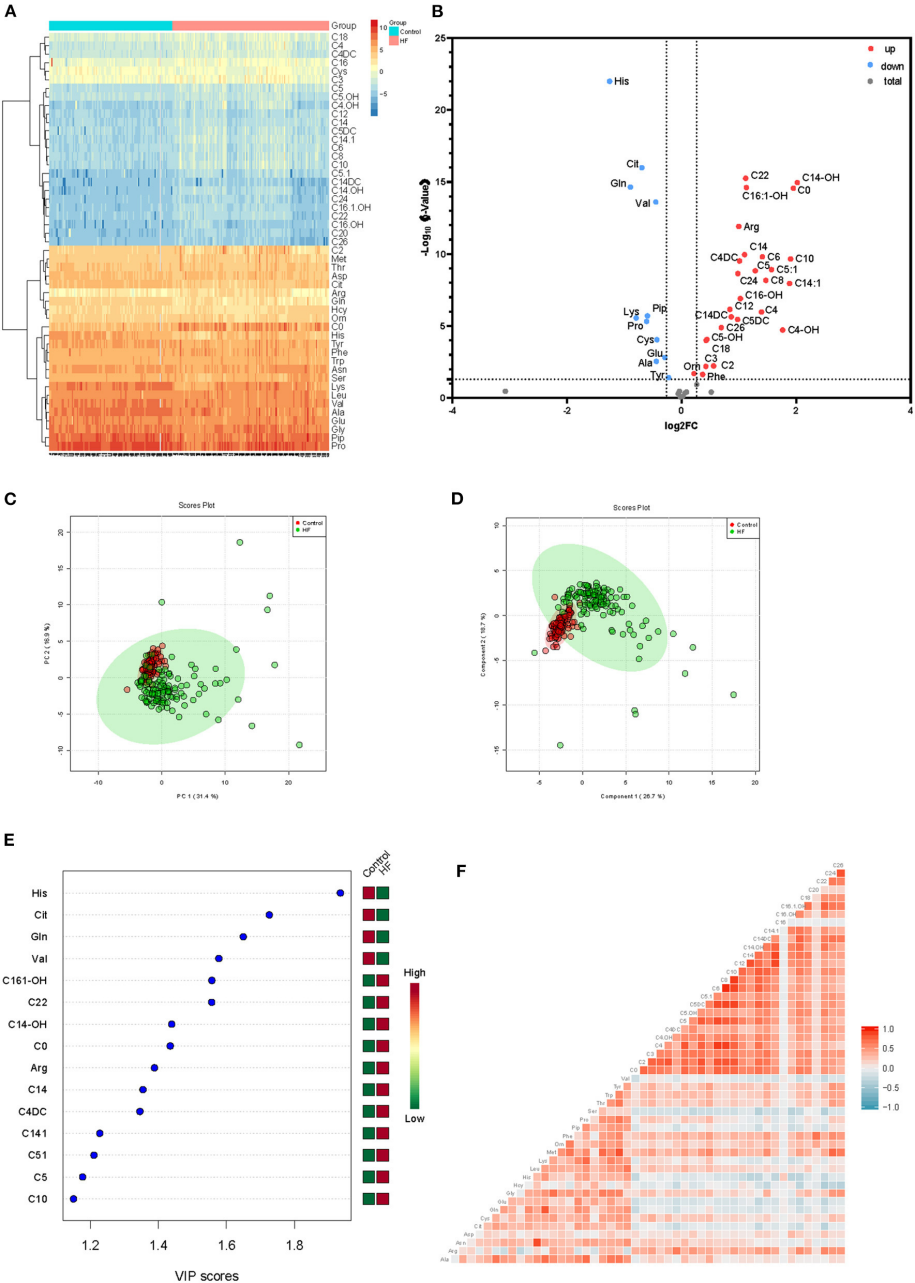
**(A)** Heatmap of significantly differential metabolites between HF patients and control group, depicting the levels of metabolites in each sample and the variation in each metabolite between samples, ranging from blue (low intensity or downregulation) to yellow and red (high intensity or upregulation). **(B)** Volcano plot for significantly differentially metabolites between the two groups. Red dots indicate that the metabolite was significantly changed in terms of the *P*-value and log_2_ fold-change value; gray circles indicate that the metabolite level was not significantly altered in terms of *P*-value. **(C)** PCA and **(D)** PLS-DA analysis of 49 metabolites, the green dots represent HF patients while the red dots represent control group. **(E)** VIP scores of differential metabolites. **(F)** Correlation analysis of all metabolites, ranging from blue (low correlation) to red (high correlation).

**Figure 3:**
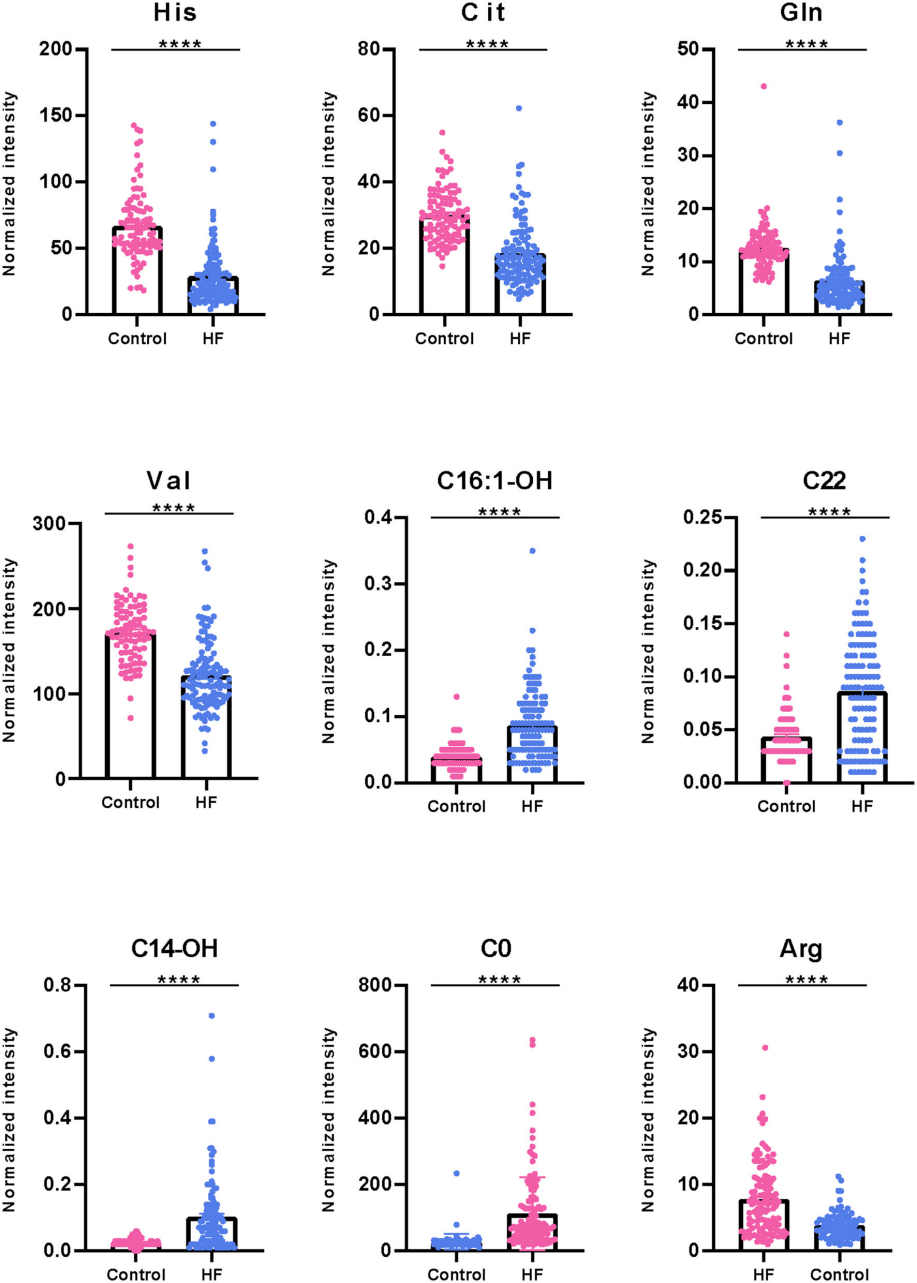
Boxplot of significantly differential metabolites between HF patients and control group. Some representatively differentiated metabolites with *P* < 0.05 were showed (****P < 0.0001).

To explore the metabolic differences between HF patients and the control group, the study measured 49 metabolites including 23 amino acids and 26 carnitines. As is shown in Figure 2A; Supplementary Figure 1, a heap map with hierarchical clustering distinguished that some metabolites were significantly different between HF patients and the controls. These included medium-chain acylcarnitine [decanoyl-carnitine (C10), tetradecenoyl-carnitine (C14:1)], long-chain dicarboxyl-acylcarnitine [hydroxytetradecanoyl-carnitine (C14-OH)], and amino acids Histidine (His) and Arginine (Arg). In the volcano diagram shown in Figure 2B, the red dots represent metabolites with significant differences (*p* < 0.05). Overall, 38 out of 49 metabolites were significantly different between the control and HF groups (Table 2), and 27 metabolites were increased in HF patients while11 metabolites were downregulated. The quantitative results of these metabolites showed a vague differentiation between HF patients and the controls, as presented in the PCA score plot (Figure 2C), but the supervised multivariate analysis of PLS-DA revealed a distinct separation between the two groups (*R*^2^ = 0.84; *Q*^2^ = 0.71; Figure 2D). This outcome suggests a profound metabolic alteration between the HF group and the control group and these metabolites may help to classify HF patients from control group effectively, which is consistent with some former studies (7, 24). The metabolites with significant differences were defined as having a Fold Change>1.5 and *P* < 0.05. Among all the different metabolites, His, Arg, Citrulline (Cit), Glutamine (Gln), Valine (Val), hydroxyhexadecenyl-carnitine (C16:1-OH), acylcarnitine (C22), hydroxytetradecanoyl-carnitine (C14-OH), and free carnitine (C0) were the most important features with respect to HF metabolism (Figure 2E). The correlations of these metabolites were detected (Figure 2F). At the same time, significant changes of these nine metabolites were identified between the two groups (Figure 3). Clearly, most carnitines were positively correlated with each other, whereas nearly half of amino acids were negatively correlated with carnitines.

**Table 2:** Differential metabolites between the Heart Failure patients and control group from LC-MS analysis.

**Metabolite**	**Heart failure**			**Control** ** (*n* = 97)**	**Log_**2**_ fold change**	***P*-value**
	**CHD** ** (*n* = 67)**	**VHD** ** (*n* = 16)**	**DCM** ** (*n* = 13)**			
C14-OH	0.11 (0.08–0.16)	0.03 (0.02–0.1)	0.03 (0.01–0.07)	0.02 (0.02–0.04)	2.02	1.10E-15
C0	81.99 (46.44–151.64)	94.18 (67.86–132.11)	64.57 (35.93–108.16)	25.97 (21.54–30.35)	1.95	2.71E-15
C10	0.21 (0.16–0.32)	0.18 (0.11–0.2)	0.125 (0.09–0.24)	0.06 (0.05–0.09)	1.90	2.20E-10
C14:1	0.2 (0.15–0.30)	0.2 (0.16–0.28)	0.14 (0.10–0.24)	0.06 (0.04–0.09)	1.89	1.10E-08
C4-OH	0.15 (0.09–0.30)	0.08 (0.05–0.17)	0.06 (0.03–0.15)	0.04 (0.03–0.05)	1.76	1.91E-05
C5:1	0.09 (0.08–0.14)	0.08 (0.06–0.08)	0.07 (0.06–0.10)	0.02 (0.02–0.04)	1.57	1.21E-09
C8	0.16 (0.11–0.23)	0.15 (0.12–0.18)	0.12 (0.08–0.20)	0.07 (0.05–0.09)	1.47	6.82E-09
C6	0.11 (0.01–0.18)	0.14 (0.09–0.21)	0.12 (0.09–0.22)	0.06 (0.05–0.08)	1.41	1.55E-10
C4	0.42 (0.29–0.92)	0.49 (0.36–0.50)	0.29 (0.12–0.54)	0.2 (0.15–0.26)	1.40	1.07E-06
C5	0.22 (0.16–0.26)	0.22 (0.15–0.35)	0.22 (0.15–0.27)	0.11 (0.08–0.14)	1.29	1.45E-09
C16:1-OH	0.09 (0.07–0.12)	0.05 (0.04–0.05)	0.045 (0.03–0.08)	0.04 (0.03–0.05)	1.13	2.41E-15
C22	0.01 (0.08–0.13)	0.04 (0.03–0.09)	0.03 (0.02–0.07)	0.04 (0.03–0.05)	1.12	5.52E-16
C14	0.12 (0.09–0.16)	0.13 (0.12–0.15)	0.17 (0.10–0.22)	0.06 (0.05–0.08)	1.10	1.11E-10
C16-OH	0.05 (0.03–0.07)	0.04 (0.03–0.06)	0.03 (0.03–0.04)	0.02 (0.02–0.03)	1.03	1.26E-07
C4DC	0.47 (0.39–0.62)	0.23 (0.13–0.31)	0.22 (0.19–0.43)	0.21 (0.17–0.29)	1.01	3.06E-10
Arg	7.87 (6.36–10.27)	2.45 (2.26–8.5)	2.82 (2.34–4.87)	3.51 (2.69–4.81)	1.00	1.22E-12
C24	0.06 (0.05–0.07)	0.04 (0.02–0.07)	0.03 (0.02–0.07)	0.03 (0.03–0.04)	0.98	2.29E-09
C5DC	0.155 (0.09–0.24)	0.11 (0.09–0.13)	0.10 (0.06–0.16)	0.09 (0.05–0.12)	0.98	3.56E-06
C14DC	0.05 (0.04–0.06)	0.04 (0.01–0.05)	0.02 (0.01–0.03)	0.03 (0.02–0.03)	0.87	2.31E-06
C12	0.08 (0.07–0.11)	0.1 (0.07–0.17)	0.09 (0.07–0.17)	0.05 (0.04–0.07)	0.85	6.96E-07
C26	0.04 (0.03–0.06)	0.02 (0.01–0.05)	0.02 (0.01–0.08)	0.03 (0.02–0.04)	0.69	1.30E-05
C2	15.34 (7.01–21.77)	22.56 (5.22–31.27)	7.98 (5.54–14.98)	12.39 (10.62–14.86)	0.56	5.95E-03
C18	0.52 (0.39–0.72)	0.59 (0.35–1.02)	0.57 (0.46–0.87)	0.45 (0.35–0.56)	0.44	8.62E-05
C5-OH	0.21 (0.14–0.28)	0.14 (0.11–0.16)	0.12 (0.1–0.21)	0.14 (0.11–0.18)	0.43	1.00E-04
C3	2.15 (0.95–3.38)	1.93 (0.81–2.41)	1.41 (0.82–1.91)	1.32 (1.07–1.78)	0.42	6.45E-03
Phe	49.83 (40.45–72.06)	34.47 (27.54–60.12)	42.67 (27.54–60.12)	41.98 (37.63–50.17)	0.37	2.31E-02
Orn	10.655 (7.55–15.60)	8.93 (6.63–12.43)	114.07 (11.95–19.98)	10.64 (9.50–12.67)	0.22	2.04E-02
Tyr	40.05 (31.34–59.24)	40.83 (29.01–50.61)	62.98 (38.27–77.40)	65.07 (55.01–74.89)	−0.23	3.85E-02
Glu	93.02 (66.1–110.91)	110.45 (105.07–162.45)	138.28 (111.59–179.66)	147.95 (132.59–171.07)	−0.29	1.55E-03
Cys	1.48 (0.94–1.97)	1.34 (1.02–1.94)	1.04 (0.89–1.41)	1.75 (1.02–2.45)	−0.43	8.92E-05
Ala	124.85 (96.58–166.15)	82.32 (61.00–117.15)	109.16 (74.45–186.78)	198.64 (172.34–240.34)	−0.44	2.84E-03
Val	122.68 (94.91–138.58)	90.81 (72.64–98.72)	123.48 (111.38–143.98)	170.10 (148.22–193.04)	−0.45	2.48E-14
Pip	122.63 (90.13–166.96)	251.28 (195.3–281.88)	274.98 (204.52–333.02)	331.33 (263.92–426.04)	−0.59	1.98E-06
Pro	341.17 (158.78–395.71)	312.75 (230.74–347.64)	350.60 (299.31–473.51)	545.58 (433.47–718.71)	−0.61	4.80E-06
Cit	14.49 (11.44–19.55)	15.62 (12.27–16.38)	20.76 (17.05–33.98)	29.59 (24.03–35.18)	−0.69	1.02E-16
Lys	79.21 (38.61–120.74)	64.3 (58.63–133.78)	68.86 (46.58–96.80)	176 (126.53–209.57)	−0.79	2.78E-06
Gln	5.99 (4.53–8.18)	4.51 (3.14–4.88)	6.14 (3.73–8.21)	12.11 (10.63–14.47)	−0.89	2.27E-15
His	19.22 (11.03–35.44)	17.19 (14.83–21.07)	117.34 (109.67–147.84)	61.95 (51.83–79.14)	−1.25	1.02E-22

*Values are reported as median (interquartile range)*.

*Ala, Alanine; Cys, Cysteine; Lys, Lysine; Glu, Glutamine; Pip, Piperine; Pro, Proline; Phe, Phenylamine*.

### Characteristic Changes Between CHD and VHD Metabolite Profiles

**Figure 4:**
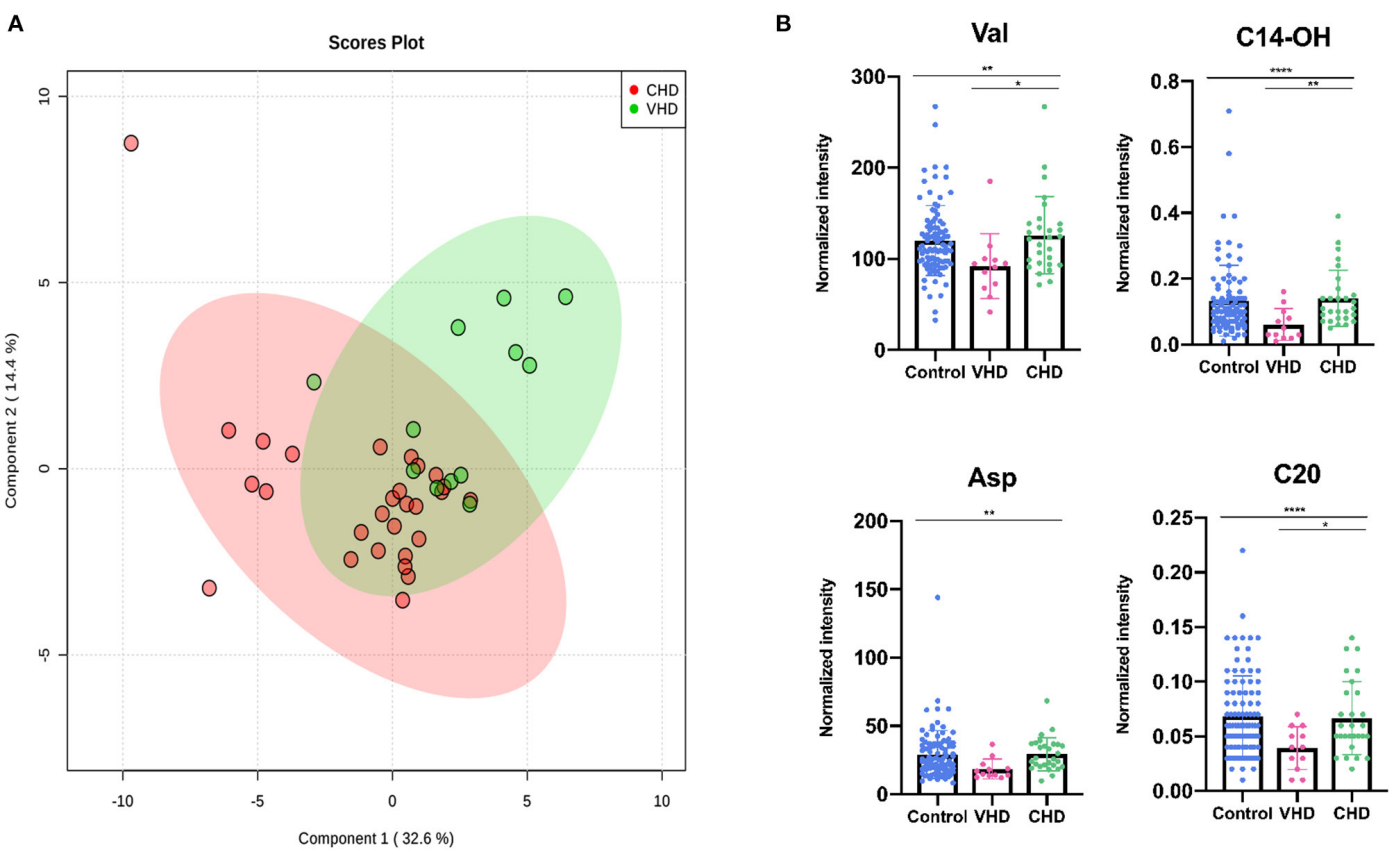
Diagnostic effectiveness of differential metabolites between HF patients and control group. **(A)** The receiver operating characteristic (ROC) curve of significantly changed metabolites (AUC > 0.8 were showed). **(B)** Correlation matrix of these differential metabolites. *p < 0.05; **p < 0.01; ***p < 0.001; ****p < 0.0001.

For a more detailed view of changes between CHD and VHD patients, metabolite concentrations were measured among the participants. Patients with left ventricular ejection fraction (LVEF) >35% were excluded. As shown in Figure 4A, the distribution of metabolic phenotypes in the CHD group was relatively concentrated. Additionally, there exhibited a tendency in the classification of metabolic phenotypes between the CHD and VHD groups. Meanwhile, the differences between the metabolites were evaluated. Val, C14-OH, Asp, and eicosyl-carnitine (C20) presented as significantly different between the two groups (*P* < 0.05) (Figure 4B), illustrating that the extent to which these metabolites were changed in CHD was different from their VHD counterparts compared with the control group.

### Characteristic Changes Between CHD and DCM Metabolite Profiles

**Figure 5:**
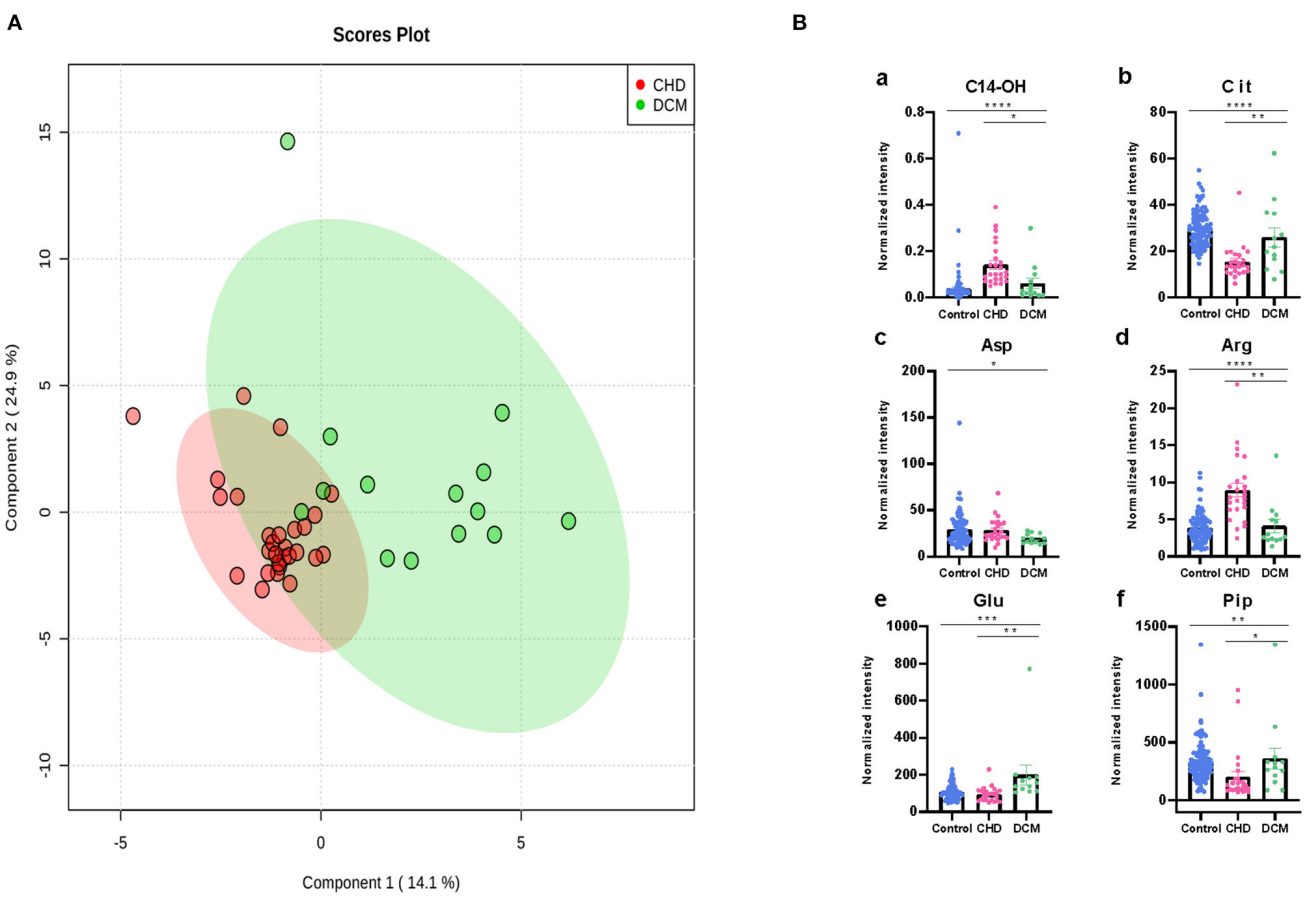
Differential metabolites differentiate patients with CHD from patients with VHD. **(A)** PLS-DA analysis of 49 metabolites between CHD and VHD patients. The green dots represent VHD patients while the red dots represent VHD patients. **(B)** Asp, Val, C14-OH, C20 changed significantly in VHD and CHD patients (*P < 0.05, **P < 0.01, ***P < 0.001, ****P < 0.0001).

The metabolic changes between CHD and DCM were analyzed. Patients with left LVEF%>35% were excluded. The CHD cohorts were separated from the DCM cohorts in the two-dimensional score plots (Figure 5A). Similarly, we performed one-way ANOVA between all the differential metabolites mentioned in the previous section, discovering that C14-OH, Cit, Arg, Aspartic acid (25), Glutamine acid (26), and Piperine (Pip) changed more drastically in DCM than in CHD (Figure 5B).

### Sensitivity and Specificity of HF Related Metabolites

**Figure 6:**
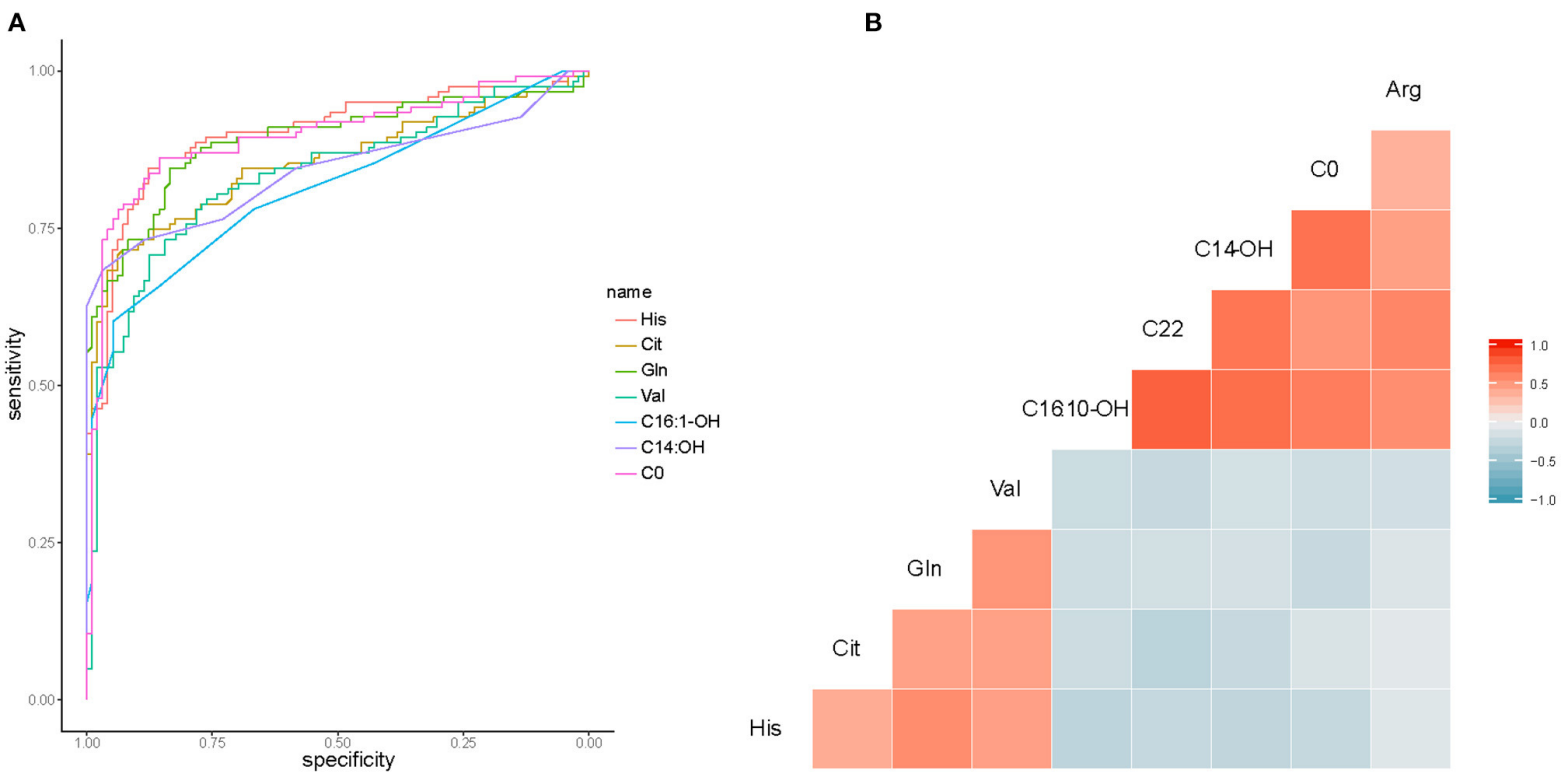
Differential metabolites differentiate patients with CHD from patients with DCM. **(A)** PLS-DA analysis of 49 metabolites between CHD and DCM patients. The green dots represent DCM patients while the red dots represent CHD patients. **(B)** Asp, Arg, Cit, Glu, Pip, C14-OH, changed significantly in CHD and DCM patients. (*P < 0.05, **P < 0.01, ***P < 0.001, ****P < 0.0001).

The top 9 candidates associated with HF were further evaluated for their capacity to differentiate control from HF patients. To estimate the AUC value, we performed ROC analysis. In this analysis, we observed good sensitivity and specificity. The AUC values of His, Cit, Gln, Val, C16:1-OH, C14-OH, and C0 were 0.90, 0.85, 0.89, 0.82, 0.81, 0.84, and 0.90, respectively (Figure 6A). C22 and Arg were not shown in the picture (AUC <0.8). Metabolite correlations were also evaluated. It was apparent that amino acids were negatively correlated with carnitine (Figure 6B).

Additionally, we examined the significantly changed metabolites between CHD and VHD, as well as between CHD and DCM. ROC analysis showed that AUC values are 0.77 for Val, 0.80 for C14-OH, and 0.78 for Asp between CHD and VHD (Supplementary Figure 2A). Furthermore, AUC values are 0.83 for C14-OH, 0.75 for Asp, 0.74 for Cit, 0.87 for Arg, 0.89 for Glu and 0.77 for Pip between CHD and DCM (Supplementary Figure 2B).

### Correlation of Metabolites With Clinical Features

**Figure 7:**
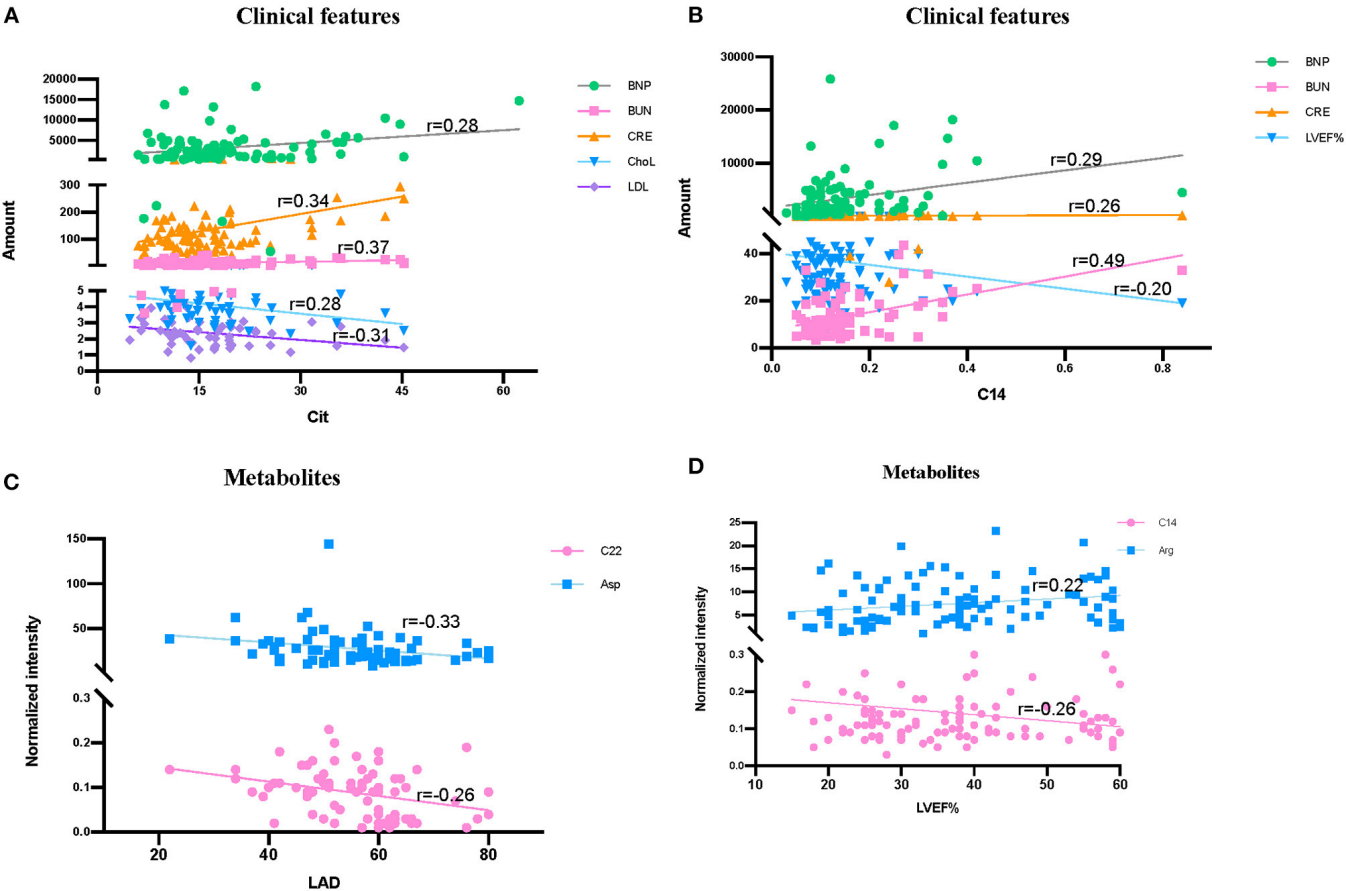
Correlation among clinical features and significantly differentially metabolites in HF patients and control groups. **(A)** Correlation among Cit, clinical renal function features (BNP, BUN, and CRE) and serum lipids index (ChoL and LDL). **(B)** Correlation among C14, LVEF% and clinical renal function features (BNP, BUN, and CRE). **(C)** Correlation among LAD, C22, and Arg. **(D)** Correlation among LVEF%, C14 and Arg. Data were analyzed by linear regression in figure.

The correlation of metabolites with clinical features LVD, LVEF%, index of serum lipids (Chol, TG, HDL, and LDL), and index of renal function (BNP, BUN, CRE, and CysC) were tested (Figure 7). Significant correlations were found among Cit and several clinical features, including BNP (*r* = 0.28), BUN (*r* = 0.37), CRE (*r* = 0.34), Chol (*r* = −0.28), and LDL (*r* = −0.31) (Figure 7A). At the same time, C14 had a strong correlation with BUN (*r* = 0.49), while it had a weak correlation with BNP (*r* = 0.29), CRE (*r* = 0.26), and LVEF% (*r* = −0.20) (Figure 7B). Additionally, left atrium diameter (LAD) had a weak correlation with C22 (*r* = −0.33) and Asp (*r* = −0.26) (Figure 7C), while LVEF% had a weak correlation with Arg (*r* = 0.22) (Figure 7D).

## Discussion

HF, which is defined as a myocardial derangement causing systolic and/or diastolic ventricular dysfunction, is based on the abnormal metabolism of myocardial substrate and the disturbance of energy metabolism ischemia or hypoxia (1, 7, 10, 27). Metabolomics, which measures chemistry and represents an integrated readout of upstream genetic, transcriptomic, and proteomic variation, has developed as a powerful tool for understanding of physiology or pathophysiology of chronic diseases (28–30). The analysis of carnitines and amino acids in dry blood spots by LC/MS is an easy-to-complete and robust method for the clinic. Only several microliters of blood samples are needed, and the data acquisition can be finished in a few minutes. Thus, it could be a simple way for the patients with cardiovascular diseases to monitor the development of these diseases. Using this targeted, quantitative metabolic profiling method, we successfully identified a group of circulating metabolites that significantly increased in HF group when compared to non-HF controls. These metabolites, including long-chain dicarboxyl-acylcarnitine (C14-OH, C16:1-OH) and docosanoylcarnitine (C22), which were significantly upregulated in HF compared with the controls (Figure 2), consistent with previous studies (31, 32). Inside the cells, fatty acids are activated by different long-chain acyl-CoA synthetases, which are specific for various sized fatty acids, into long-chain acyl-CoAs (33, 34). Then, they are transported across the mitochondrial membrane through the mitochondrial carnitine–acylcarnitine cycle for β-oxidation (35, 36). Plasma long-chain acylcarnitine elevation in HF patients reflected impaired or dysregulated fatty acid oxidation and mitochondrial dysfunction, which have both been recognized as potential therapeutic targets in HF (7, 36, 37). Additionally, the significantly lower concentrations of several tricarboxylic acid (TCA) related intermediates, such as Ala, Cys, Lys, His, Gln, Tyr, and Val (Figures 2A, 3), likely contributed to impaired mitochondrial metabolism. Moreover, LAD was negatively correlated with C22 (*r* = −0.33) between HF and control groups (Figure 7C). All these findings illustrate the distinct metabolic profile in HF patients compared with the control group.

Arg is a critical physiologic precursor for nitric oxide (NO) production and a key modulator of vascular tone whose ratio (asymmetric dimethylarginine/symmetric dimethylarginine) functions to monitor pathophysiological states in cardiovascular diseases (38–41). In our results, Arg and its related metabolic pathway also acted as an important role in cardiovascular diseases. At the same time, it was reported that high symmetric dimethylarginine levels were associated with significantly obstructive coronary artery disease (42). Arg can convert to Cit and NO by NO synthase in endothelial cells (43, 44). To this end, ample evidence indicates that atherogenic oxidized low-density lipoprotein (ox-LDL) can inhibit endothelial-derived NO, thus leading to leukocyte deposition, platelet adhesion, platelet aggregation, and subsequent release of vasoconstrictor species such as serotonin and TXA2 (45–48), which lead to endothelial-dependent vasomotor abnormalities (49). Inflammatory and immunologic reactions are key factors in the process of atherogenesis, which may result from deposition of leukocytes, especially T lymphocytes and macrophages (50). It has been confirmed that ox-LDL reduces NO production by disrupting Arg–NO metabolism. Anti-oxidants and lipid lowering agents could benefit the cardiovascular systems partly due to restoration of Arg transport (35, 51–53). Glu is a precursor of Orn, which can be converted to Cit by the intestine. In our study, Cit was significantly reduced in HF patients (Figure 3), whereas Arg intensity was significantly elevated. Additionally, Cit was negatively correlated with LDL (*r* = −0.31), and LVEF% was negatively correlated with Arg (*r* = 0.22) in a dose-dependent manner (Figure 7A). As observed in the metabolite profile between CHD and DCM, Arg in the DCM group dramatically decreased (Figure 5B). It was almost half of the intensity compared with control group, while its intensity in CHD patients was distinctly higher than DCM patients. Cit and Glu were elevated significantly in the DCM group, but slightly decreased in the CHD group compared with the control group (Figure 5B).

It is worth noting that Cit is positively correlated with urea cycle metabolites BNP (*r* = 0.28), BUN (*r* = 0.37), and CRE (*r* = 0.34) (Figure 7A). Cit is released in the portal vein due to the lack of arginino-succinate synthase in enterocytes (53, 54). Under normal conditions, only small net Cit uptake by the liver enters systemic circulation and is then transformed in the kidney to Arg. The increased LDL in plasma means more ox-LDL was produced, inhibiting the production of NO and reducing Cit (Figure 5B) in an internally conservative cycle, while disturbing Arg–NO metabolism and resulting in excess Arg. Given the strong relationship between renal dysfunction and cardiovascular diseases (55, 56), it is suggested that Arg derived ureagenesis was activated, which leads to the decrease in urea cycle metabolites BNP, BUN, and CRE and decreased pre-hepatic conversion of Arg to Cit (53).

Abundant evidence has demonstrated that DCM, characterized by left ventricular or biventricular dilation and impaired contraction, is the result of heart remodeling and fibrosis (2, 57, 58). In adults, guidelines recommend implantable cardioverter-defibrillator (ICD) placement for primary prevention in patients with DCM and an ejection fraction of 35% after a 3-month waiting period with directed medical therapy (59). Therefore, we compared metabolic data of DCM patients with CHD patients (LVEF% ≤ 3 5%). Notably, CHD and DCM patients exhibited similar changes in amino acid metabolism compared with control group (Figure 5B), but changes in CHD patients were more significant. Cit, Asp, Arg, Glu, and Pip were among the top selected metabolites, showing the strong association of plasma amino acid with HF causes. Biochemically, amino acids can form precursors of glucose and FA metabolism through deamination or transamination and participate in metabolic pathways, such as the TCA cycle. In addition, amino acid-derived bioactive substances play an important role in the cardiovascular system (60). In HF, myocardial remodeling is obvious, and cardiac function is often difficult to reverse, which is confirmed by our metabolites results. Heart remodeling is characterized by markedly increased fibrosis in the matrix (61). Pip has been reported to decrease the phosphorylation of extracellular signal-regulated kinase (ERK) (62) and activate AMPKα signaling (63, 64), as well as attenuate cardiac fibrosis and protect against cardiac hypertrophy (65). Pip was markedly decreased in the CHD group while only slightly decreased in DCM group, indicating that cardiac fibrosis and myocardial remodeling, which may result from ischemic damage or myocardial infarction, were more serious in CHD.

Either pathological remodeling of the valve tissues or secondary consequences of a change in the anatomy and geometry of the cardiac structures will result in VHD (4, 66). Short course VHD, including aortic stenosis, mitral regurgitation, aortic regurgitation, mitral stenosis or endocarditis, can be quickly controlled and heart function can be reversed if percutaneous mitral valve repair, transcatheter aortic valve implantation or surgery is displayed in time (67). Compared with CHD patients, VHD patients had a significantly different metabolic profile (Figure 4A), which showed a sharp decrease in Val, Asp, C20, and C14-OH (Figure 4B).

The different potential causes of HF, such as CHD, VHD, and DCM, and the often-slow progression from initiation of cardiac damage to eventual signs and symptoms of HF make medical intervention in early stages to prevent irreversible damage particularly challenging. In this study, His, Cit, Gln, Val, C16:1-OH, C14-OH, and C0 were sensitive (AUC>0.8) in differentiating HF patients from the control group (Figure 6A). At the same time, C14-OH and Asp were good enough for identifying CHD from VHD (Supplementary Figure 2A). Moreover, Arg, Glu, and C14-OH (AUC>0.8) could effectively distinguish CHD and DCM (Supplementary Figure 2B).

This study investigated the role of targeted metabolomics in relation to different causes in patients with HF. The metabolic profile in patients with DCM and VHD were altered in comparison to patients with CHD. Therefore, metabolic signature was a marker for disease diagnosis in addition to LVEF%, left ventricular end-diastolic diameter (LVEDD), and left ventricular end-systolic diameter (LVESD). This also suggests that functional recovery of the heart is reflected in the metabolic signature. From the metabolic analysis, CHD-, VHD-, and DCM- induced HF all shared insufficient supply of cardiac capacity. However, VHD is characterized by abnormal regulation of glycolipid metabolism, while CHD hypoxia reflects major metabolic changes, and DCM shared similar metabolic changes with CHD but was closer to the control group than CHD in its metabolite profile. These metabolic alterations, including amino acid (Val, Asp, Cit, Arg, Glu, and Pip) and carnitine (C14-OH, C16:1-OH, C22, and C20) alterations, imply potential treatment targets. For example, Cit supplementation seemed to improve cardiac function in patients with preserved ejection fraction or HF (68–71). Better understanding of the metabolome facilitates the therapeutic development of new targets for HF (72).

Myocardial remodeling is an important pathological factor leading to the development of HF, at present, imaging examinations such as echocardiography and nuclear magnetic resonance can only evaluate the remodeling of the heart structure, but not the molecular level. For patients with HF, after standard anti-heart failure drug treatment or device treatment, may experience improvement in ventricular function and/or structural recovery, which is called “reverse remodeling” (73–75). Studies have shown that beneficial changes in molecular, metabolic, and extracellular matrix (ECM) properties of the myocardium are closely related with ventricular reverse remodeling (73, 75, 76). The results of this article show that the degree of abnormal myocardial metabolism is different between different types of cardiomyopathy, which is helpful to evaluate heart function from the perspective of metabolic remodeling.

## Limitation

There are several limitations for the present study. Firstly, we used only one-time-point plasma samples instead of samples collected from a time course, the latter of which are much more difficult to obtain. Thus, the identified metabolic changes among HF patients (CHD, VHD, and DCM) cannot be firmly confirmed as effective early diagnostic tools to monitor heart conditions. Secondly, our study reported the metabolic phenotype differences among HF groups; however, more clinical or elementary experimental studies about the prognosis effects of these phenotypes and differential metabolites in the pathogenesis remains to be clarified in the future. Thirdly, the patients were enrolled from a single center and each group had a relatively small sample size. Our future studies will search for multi-center cooperation to collect a larger number of clinical samples as external validation cohort.

## Conclusion

In conclusion, rapid diagnosis of CHD, VHD, and DCM metabolite profiles could be achieved by using a simple LC/MS approach, which only needs a small spot of blood and is easy to collect. These may lead to a future home-based medical service for people with HF for monitoring heart condition and underlying disease processes.

## Data Availability Statement

The raw data supporting the conclusions of this article will be made available by the authors, without undue reservation.

## Ethics Statement

The study was approved by the ethics committee of the first affiliated hospital, Dalian medical university. The patients/participants provided their written informed consent to participate in this study.

## Author Contributions

CL: data acquisition, analysis, and writing original draft. RL: data acquisition. YL: data interpretation. ZL: article revising. YS: data analysis. PY: conceptualization, writing—reviewing, and editing. RH: conceptualization and revising. All authors: contributed to the article and approved the submitted version.

## Conflict of Interest

The authors declare that the research was conducted in the absence of any commercial or financial relationships that could be construed as a potential conflict of interest.
