# Supplementary material for: Characteristics of Blood Metabolic Profile in Coronary Heart Disease, Dilated Cardiomyopathy and Valvular Heart Disease Induced Heart Failure
Source: Front Cardiovasc Med. 2021 Jan 20;7:622236. doi: 10.3389/fcvm.2020.622236 (PMC7856915; doi:10.3389/fcvm.2020.622236)
Supplement: Supplementary file 1 [file Table_1.DOCX]

Supplementary Material

# Supplementary Data

None.

# Supplementary Figures and Tables

## Supplementary Figures

**Supplementary Figure 1.** Heatmap of significantly differential metabolites between HF patients and control group. Metabolites with the absolute log2 fold change value greater than 1.2 are included.

**Supplementary Figure 2.** Diagnostic effectiveness of differentiated metabolites. (A) The receiver operating characteristic (ROC) curve of significantly changed metabolites between CHD and VHD. (B) The ROC curve of significantly changed metabolites between CHD and DCM.
